# Editorial Correspondence

**Published:** 1877-07

**Authors:** Wm. Lewis Dinkins

**Affiliations:** Louisville, Ga.


### EDITORIAL CORRESPONDENCE.

Louisville, Ga., July 5, 1877.

*Editors Atlanta Medical and Surgical Journal:*

Deeming it the duty of physicians to report all cases of importance, as well as being afforded pleasure "to add one cubic inch to the stature," I take this opportunity of reporting to your generous pages the operation for and treatment of a case of strangulated femoral hernia. In so doing, I would offer but this apology—that though this form is rare in the male, circumstances of a peculiar nature rendered the full and complete recording of all the points impossible. Should the manner in which this is written betray the fact that I am not a Solomon in the science, I would tell you that I am an unassuming country Doctor from the *wire-grass and whortleberry* portion of Georgia, content with minor operations in surgery—such as cutting off of dog's tails in this season, that they may not in the persuit of the rabbit knock off the whortleberrys. But to the case. Mr. A. J. Hudson, aged 66, weight about 160 pounds, known to be temperate and in good health, was suddenly attacked on January 20th, with violent symptoms (resembling those in colic) after having drank freely of cold water when very warm. Found in his room by Mrs. Hudson, while thus under the false impression that he was only suffering from an attack of colic, every persuasion was instituted by Mrs. H. to have the family physician summoned. Before sending for the physician, however, recourse was had to remedies whose medical efficacy seemed most adequate to the demand in the case. Camphor given internally, with opium by the mouth, and enemas substantially constituted the treatment by Mrs. H., who was of course very uneasy. These remedies by smoothing over the trouble, and allaying the nervous excitement produced only temporary relief. The enema emptied the bowels below the stricture. On the morning of the 21st, vomiting set regularly in, with other symptoms which led to the discovery of an enlargement in the right groin. Becoming alarmed, Mrs. Hudson again expressed a desire that the Doctor be called in. Dr. Pilcher, my friend and colleague, living six miles distant was sent for. After arriving and making a thorough examination, he diagnosed the case, femoral hernia. Knowing the impropriety, as well as the injury of attempting to give relief by the administration of medicine, an operation was advised as the only means of relief. Having many cases on hand, just at this time that demanded his attention—with the failure to get assistants when desired, the time for operation could not be agreed upon till the 25th, much to the injury and suffering of the patient, as well as to the displeasure of the attending physician. During this interval incessant vomiting, (which was very offensive) considerable fever, high bounding pulse and hiccough were among the unfavorable symptoms. From the time of the injury to the operation, which was five days, no nourishment was taken, no action upon the bowels, (save that from below the stricture) was had. On the morning of the 25th, Dr. Pilcher and myself held a consultation and decided in the face of every unfavorable symptom to give the patient the chance of recovery. Taxis had been resorted to, but reduction by this means failed. Before attempting reduction by taxis the abdominal viscera was relaxed by flexing and adducting the thigh—thus opening up the saphenous canal. Now for the operation. Drs. Thompson, Stewart and Oliphant being present, assisted in the performance of the operation. A table of the proper length was procured and brought forward, the patient placed upon it and thoroughly anæsthetized by chloroform and sul. ether in proportion of one part of the former and two of the latter. The bladder having been looked after and the parts that were the seat of operation shaved—the attending physician, Dr. Pilcher, commenced in good earnest, by cutting down in the direction of the sac—gently exploring in front of the knife with his finger and probe—attempting to detect the pulsations or presence of blood vessels in the vicinity of the sac. The incision was made from an inch above to an inch below the sac. This was not done according to Erichsen, by pinching up a fold of skin—introducing the knife through its base with the back of the instrument turned towards the hernia, and then cutting upwards, but the incision was made down upon the parts through the fascia till coming close upon the sac. As tissue after tissue was arrived at the hook was used to pinch up the parts—the groove inserted—and the tissues cut down upon and laid aside. When coming down to the sac a halt was made to examine thoroughly if the gut had been reached, but after examination by all present, the incision was made through the sac and the gut carefully exposed. We did not, as is usually the case, find a layer of fluid between the sac and gut. This somewhat nonplused us, and we thought things were not progressing favorably. The intestine protruded fully two inches, and upon percussion showed it contained fecal matter. The intestine was very dark and had the appearance of a mortified part. The sac having been carefully examined as before said, a portion of it was seized between the finger and thumb—an opening was made with the back of the knife to the gut. The gut having been thoroughly exposed, and notwithstanding its dark appearances was thought best to reduce it and trust to nature for a restoration of its vitality. The stricture was removed by making an incision at the junction of Ginbernat's or third attachment of Poupart's ligament. The intestine was placed in its normal position, the wound thoroughly cleansed of blood, and stitches taken externally. There was no difficulty attending the administration of the anæsthetic, nor was it followed by any unusual sickness of stomach; slight depression followed its administration. The vomiting with regurgitation from the bowels seemed greatly augmented by the shock given to the nerves of the bowels by the operation. Indeed the depression consequent upon this extreme nausea was alarming, and our patient, who had undergone a most beautiful operation, seemed moribund. After almost contiual vomiting for thirty hours after the operation, the patient exhausted and relaxed dozed into a semi-sleep condition. The bowels at this period from relaxation—as well as from irritability caused by the retention of old excrement—commenced and continued to act—notwithstanding the administration of opium, tannin, bismuth, etc. On the morning of the 28th, large doses of bismuth, sub. nit., opium, tannin and chalk were commenced and given till noon, when the bowels were checked. Brandy in sweet milk and sugar was given whenever the case demanded it. The most nutritious articles of diet were used. The tongue becoming dry, the urine scanty, turpentine emulsion was administered; quinine in tonic doses was used.

July 1st.—Patient brighter than at any time since the operation, bowels acted once in the last twenty-four hours, slight congestion of lungs from lying upon the back, clear of fever. The wound doing well.

Monday, July 2d.—Patient still improving in strength and appetite, bowels moved once in twenty-four hours, actions consistent pulse regular, no fever, some cough.

Tuesday, July 3d.—Treatment—quinine in grain doses to act as a tonic, equal parts paregoric and squills, honey and ipecac, as an expectorant to relieve slight cough. Patient sitting up and perfectly clear of fever, wound healing rapidly—patient discharged.

In conclusion, I would say that the points of interest to us in this case, are, the unusual occurrence of this form of hernia in the male, and the recovery after so long standing strangulation without the loss of any portion of the bowel.

WM. LEWIS DINKINS, M.D.

